# Calenduloside E Ameliorates Myocardial Ischemia-Reperfusion Injury through Regulation of AMPK and Mitochondrial OPA1

**DOI:** 10.1155/2020/2415269

**Published:** 2020-08-31

**Authors:** Min Wang, Rui-ying Wang, Jia-hui Zhou, Xue-heng Xie, Gui-bo Sun, Xiao-bo Sun

**Affiliations:** Beijing Key Laboratory of Innovative Drug Discovery of Traditional Chinese Medicine (Natural Medicine) and Translational Medicine, Institute of Medicinal Plant Development, Chinese Academy of Medical Sciences & Peking Union Medical College, Beijing 100193, China

## Abstract

Calenduloside E (CE) is a natural triterpenoid saponin isolated from *Aralia elata* (Miq.) Seem., a well-known traditional Chinese medicine. Our previous studies have shown that CE exerts cardiovascular protective effects both *in vivo* and *in vitro*. However, its role in myocardial ischemia/reperfusion injury (MIRI) and the mechanism involved are currently unknown. Mitochondrial dynamics play a key role in MIRI. This study investigated the effects of CE on mitochondrial dynamics and the signaling pathways involved in myocardial ischemia/reperfusion (MI/R). The MI/R rat model and the hypoxia/reoxygenation (H/R) cardiomyocyte model were established in this study. CE exerted significant cardioprotective effects *in vivo* and *in vitro* by improving cardiac function, decreasing myocardial infarct size, increasing cardiomyocyte viability, and inhibiting cardiomyocyte apoptosis associated with MI/R. Mechanistically, CE restored mitochondrial homeostasis against MI/R injury through improved mitochondrial ultrastructure, enhanced ATP content and mitochondrial membrane potential, and reduced mitochondrial permeability transition pore (MPTP) opening, while promoting mitochondrial fusion and preventing mitochondrial fission. However, genetic silencing of OPA1 by siRNA abolished the beneficial effects of CE on cardiomyocyte survival and mitochondrial dynamics. Moreover, we demonstrated that CE activated AMP-activated protein kinase (AMPK) and treatment with the AMPK inhibitor, compound C, abolished the protective effects of CE on OPA1 expression and mitochondrial function. Overall, this study demonstrates that CE is effective in mitigating MIRI by modulating AMPK activation-mediated OPA1-related mitochondrial fusion.

## 1. Introduction

Myocardial ischemia/reperfusion injury (MIRI), which occurs due to blood restoration after a critical period of coronary artery obstruction, causes increased myocardial dysfunction and further cardiomyocyte death, in particular myocardial infarction [1]. Currently, bedside therapies to mitigate ischemia/reperfusion- (I/R-) mediated myocardial damage remain limited largely due to the complex mechanisms that contribute to I/R.

*Aralia elata* (Miq.) Seem. (AS) is traditionally used as a tonic for antiarrhythmic, antiarthritic, antihypertensive, and antidiabetic purposes in traditional Chinese medicine [2]. The total saponins of AS were developed as a new drug called *A*. *elata* Xinmaitong capsules for the treatment of coronary heart disease which has successfully completed phase III clinical trials in China [2]. Calenduloside E (CE) is one of the major natural pentacyclic triterpenoid saponins present in a wide variety of Aralia plants [3]. A number of studies indicate that CE possesses considerable cardiovascular regulatory effects. We previously demonstrated that CE exerts an antiarrhythmic effect [4] and protects against oxidative stress-induced injury in cardiomyocytes [5]. We also found that CE protects endothelial cells from injury and reduces apoptotic endotheliocytes [6, 7]. However, the protective effect of CE on myocardial I/R (MI/R) injury and its underlying mechanism are still not fully understood.

Mitochondria are enriched in cardiomyocytes and have been considered key organelles for controlling cardiac function and cardiomyocyte viability via constant fission and fusion under physiological and pathological conditions [8]. Normal mitochondrial fission promotes mitochondrial redistribution, while mitochondrial fusion enhances the supplementation of mitochondrial components and keeps the mitochondrial network in a healthy state. The imbalance of mitochondrial fission and fusion, which results in mitochondrial dysfunction, plays a crucial part in the pathogenesis of MI/R injury [8]. Excessive mitochondrial fission and reduced mitochondrial fusion have been shown to lead to the activation of apoptotic cell death during MI/R injury. Accordingly, several strategies that strengthen the mitochondrial fusion or inhibit excessive mitochondrial fission have been reported to preserve mitochondrial function of the heart against MI/R injury [9, 10]. Therefore, targeting the proteins that regulate mitochondrial dynamics is a potential strategy to prevent I/R-induced cardiac injury.

Optic atrophy 1 (OPA1), a protein embedded in mitochondrial inner membranes, primarily controls mitochondrial fusion [11]. The reduced expression of OPA1 in MI/R injury aggravates mitochondrial fragmentation and results in mitochondrial dysfunction and mitochondrial apoptosis, which contribute to the development of MI/R injury [12]. Increased OPA1 is associated with active mitochondrial fusion, and overexpression of OPA1 has been shown to reverse mitochondrial function and improve cardiac performance in the I/R heart [13]. Increasing evidence suggests that AMP-activated protein kinase (AMPK) acts as a hub to bridge mitochondrial dysfunction, and OPA1 expression is potentially regulated by the AMPK pathway [13, 14]. Thus, it is very worthwhile to explore whether CE alleviates MI/R injury by activating OPA1-related mitochondrial fusion in an AMPK-dependent manner.

In this study, we investigated the therapeutic effects of CE using a model of MI/R injury in rats and a cellular hypoxia/reoxygenation- (H/R-) induced H9c2 cardiomyocyte injury model, especially focusing on mitochondrial dynamics and function via modulating AMPK/OPA1 expression.

## 2. Materials and Methods

### 2.1. Animals

Adult male Sprague-Dawley (SD) rats weighing 230-250 g were purchased from Beijing Vital River Laboratory Animal Technology Co., Ltd., Beijing, China. The animals were housed under standard laboratory conditions (temperature of 25 ± 1°C, humidity of 50 ± 10%, and 12 h photoperiod) and allowed free access to sterile food and water. All experiments were approved by the Laboratory Animal Ethics Committee of the Institute of Medicinal Plant Development, Peking Union Medical College, and conformed to the *Guide for the Care and Use of Laboratory Animals* published by the US National Institutes of Health (NIH Publication, the 8^th^ Edition, 2011).

### 2.2. Cardiac I/R Injury Model and Treatment

The rat model of myocardial I/R was performed as described previously [15]. Briefly, rats were anesthetized with an intraperitoneal injection of sodium pentobarbital (40 mg/kg). A lead II electrocardiograph was monitored throughout the surgical process. After tracheal cannulation, a miniventilator was used to maintain gas exchange in the lungs. Following thoracotomy, the left anterior descending coronary artery was reversibly ligated with a 6-0 silk suture (except in the sham and sham+CE groups). After 30 min of ischemia, the heart was allowed 48 h of reperfusion by carefully releasing ligation. Rats were randomly divided into four groups (*n* = 12): the sham, sham+CE, I/R, and I/R+CE groups. The sham and I/R groups were administered an equal volume of ultrapure water containing 0.5% sodium carboxymethylcellulose by oral gavage for three days. In the drug treatment groups, 15 mg/kg CE was administered for three days. The last treatment was completed 60 min before vascular ligation. The concentration of CE used was selected based on our preliminary experiments.

### 2.3. Echocardiographic Assessment of Cardiac Function

Cardiac function was assessed using M-mode echocardiography according to the methods described in our previous study [16]. Briefly, rats were anesthetized with 2% isoflurane, and echocardiography was performed using a Vevo 770 high-resolution *in vivo* imaging system (FUJIFILM VisualSonics, Inc., Toronto, Ontario, Canada). M-mode tracing of the left ventricle was obtained from the parasternal long-axis view. Left ventricular ejection fraction (LVEF) and left ventricular fractional shortening (LVFS) were calculated using computer algorithms. All measurements represent the mean of 5 consecutive cardiac cycles.

### 2.4. Determination of Myocardial Infarct Size

Assessment of infarct size was determined by triphenyl tetrazolium chloride (TTC) staining, as described previously [15]. Briefly, at the indicated time point, the rat heart was sliced into five equal pieces at a line parallel to the coronary sulcus and below the ligation of the heart. The slices were then incubated with 1% TTC solution at 37°C for 15 min. Image-Pro Plus 5.0 software (Media Cybernetics, Rockville, MD, USA) was used to analyze the infarct (white) and noninfarct (red) areas after the images were captured using a high-resolution camera. The percentage of myocardial infarct was calculated as the infarct area divided by the total area.

### 2.5. Measurement of Serum Myocardial Enzymatic Levels

Blood samples were collected by abdominal aortic blood collection and centrifuged at 3600 rpm for 10 min. The serum was used for the detection of cTnI using a rat-specific ELISA kit (Beijing Expandbiotech Ltd., Beijing, China) according to the manufacturer's protocol.

### 2.6. Transmission Electron Microscopy

Isolated fresh heart tissue was fixed with 2.5% glutaraldehyde in phosphate buffer (0.1 M, pH 7.4) for 2 h at 4°C. After washing in the same buffer 5 times, the tissue samples were postfixed in 1% osmium tetroxide in 0.1 M phosphate buffer, dehydrated with a graded series of ethanol to 100%, and infiltrated with propylene oxide to embedding media (Epon 812 resin, SERVA, Heidelberg, Germany). Ultrathin sections were stained with uranyl acetate and lead citrate and observed under a JEOL JEM-1230 transmission electron microscope (JEOL Ltd., Tokyo, Japan) [17]. Digital images were analyzed using ImageJ [18] to manually generate masks of mitochondrial contours that were then used for the calculation of the mitochondrial area and total mitochondrial number.

### 2.7. Determination of ATP Content

The ATP content was determined using an enhanced ATP assay kit (Beyotime Biotechnology, China) according to the manufacturer's instructions. Briefly, 100 *μ*L of ATP detection working solution with 10 *μ*L of the supernatant sample was added to a white 96-well plate for luminescence analysis using the Tecan Infinite M1000 (Hombrechtikon, Switzerland) microplate reader. Protein concentrations were determined using a BCA protein assay kit to normalize the relative ATP content.

### 2.8. Isolation of Cardiac Mitochondria

Mitochondria were extracted from heart tissue using a tissue mitochondria isolation kit (Beyotime Institute of Biotechnology, China) according to the manufacturer's instructions. Briefly, the fresh cardiac tissue and mitochondria extraction reagent were fully mixed and stirred in a homogenizer, and the suspension was centrifuged at 1000 × *g* for 5 min at 4°C. The supernatant obtained was centrifuged at 3500 × *g* for 10 min at 4°C. The precipitate was mitochondria, resuspended in a stock solution, and stored at -80°C.

### 2.9. Cell Culture and Treatment

Rat embryonic cardiomyoblast-derived H9c2 cardiomyocytes (Cell Bank of the Chinese Academy of Sciences, Shanghai, China) were cultured in high-glucose DMEM supplemented with 10% (*v*/*v*) fetal bovine serum, 1% penicillin/streptomycin (*v*/*v*), and 2 mM L-glutamine at 37°C with 5% CO_2_ incubation. For all experiments, the cells were plated at an appropriate density in accordance with the experimental design and were grown for 24 h to reach 70% to 80% confluence before experimentation. The H/R model was built according to previously published methods [19]. Briefly, H9c2 cardiomyocytes were cultured in DMEM without glucose under hypoxia for 6 h in an anaerobic glove box (Type C, Coy Laboratory, CA, USA) and then transferred to a regular incubator with the medium replaced by a normal medium to mimic reperfusion. In the CE-treated group, H9c2 cardiomyocytes were pretreated with CE for 4 h prior to H/R. In the inhibitor-treated group, cells were preincubated with 10 *μ*M compound C (CC) for 1 h before they were treated with CE. The concentration of CC was determined based on our previous experiments [20].

### 2.10. Cell Viability Analysis

Cell viability was determined using an MTT assay, as previously described [19]. H9c2 cells were seeded at a density of 1 × 10^4^ cells/well in 96-well plates. After different treatments, the cells were incubated with 20 *μ*L of MTT (5 mg/mL) each well for 4 h. The supernatant was subsequently removed, and formazan crystals were dissolved in dimethyl sulfoxide (DMSO). Absorbance was detected at 570 nm using a microplate reader (Infinite M1000, Tecan).

### 2.11. Measurement of LDH Release

The lactate dehydrogenase (LDH) release was measured using an LDH cytotoxicity detection kit, according to the manufacturer's instructions (Nanjing Jiancheng Bioengineering Institute, Nanjing, China). Briefly, the cell medium was removed for the activity analysis of extracellular LDH, which catalyzes the conversion of lactate to pyruvate, and then reacted with 2,4-dinitrophenylhydrazine to give the brownish red color in basic solution. After the reaction, each sample was detected and the absorbance was read at wavelength 450 nm. The results are expressed as U/L.

### 2.12. Flow Cytometric Detection of the Cell Apoptosis Rate

The percentages of early apoptosis and necrosis were detected using an Annexin V-FITC/PI apoptosis kit as described in our previous study [19]. Following drug treatment, the cells were harvested, washed twice with cold PBS, and incubated in the dark with 5 *μ*L of FITC-Annexin V and 1 *μ*L of PI working solution (100 *μ*g/mL) for 15 min at room temperature. The apoptosis rate was measured using a FACSCalibur flow cytometer (BD Biosciences, CA, USA).

### 2.13. Determination of Mitochondrial Transmembrane Potential

Mitochondrial membrane potential (*ΔΨ*m) was measured using the JC-1 assay kit (Beyotime Biotechnology, China) according to the manufacturer's instructions. After treatment, cells were incubated with JC-1 (2 *μ*M final concentration) at 37°C in the dark for 30 min. Cells were then washed three times with buffer and observed using fluorescence microscopy (EVOS® FL Color, Life Technologies, Carlsbad, CA, USA). For further analysis, freshly isolated mitochondria were incubated with an equal volume of JC-1 staining solution (10 mg/mL) for 20 min at 37°C in the dark and rinsed twice with buffer. JC-1 fluorescence was measured using a microplate reader (Infinite M1000, Tecan). The green JC-1 signal was detected at excitation wavelengths of 490 nm and emission wavelengths of 530 nm. The red signal was detected at excitation wavelengths of 525 nm and emission wavelengths of 590 nm. The ratio of red and green fluorescence intensities indicated changes in the mitochondrial membrane potential.

### 2.14. Determination of Mitochondrial Permeability Transition Pore (MPTP) Opening

The MPTP opening in H9c2 cardiomyocytes was measured via calcein AM staining using a living cell MPTP fluorescence detection kit (Genmed Scientifics Inc., Shanghai, China). After treatment, the cells were washed twice with reagent A and then incubated with an intermixture of reagent B and reagent C for 20 min at 37°C in the dark. After staining, cells were washed twice with reagent A, followed by image acquisition using fluorescence microscopy (EVOS® FL Color, Life Technologies). The fluorescence intensity of the mitochondria was determined using a microplate reader (Infinite M1000, Tecan) at an excitation wavelength of 488 nm and an emission wavelength of 505 nm.

### 2.15. siRNA Transient Transfection

The siRNA against OPA1 and the negative control siRNA were purchased from Invitrogen (Thermo Fisher Scientific, Inc., Waltham, MA, USA). To transfect siRNA into cardiomyocytes, Opti-Minimal Essential Medium (Gibco, Thermo Fisher Scientific, Inc., USA) without serum or antibiotics was used to incubate the cells for 24 hours. The cells were then transfected with siRNA (20 nM) diluted by Lipofectamine RNAiMAX (Invitrogen, Thermo Fisher Scientific, Inc.) for 24 h according to the manufacturer's instructions. The knockdown efficiency of the target proteins was evaluated via western blotting.

### 2.16. Western Blotting Analysis

Heart tissues or H9c2 cells were lysed on ice with tissue or cell protein extraction reagent containing 1% phenylmethylsulfonyl fluoride. Equal amounts of protein from each sample were separated by SDS-PAGE and then transferred onto a nitrocellulose membrane. After blocking with 5% (*w*/*v*) nonfat milk powder, the membranes were incubated overnight at 4°C with appropriate primary antibodies. The primary antibodies (Abcam, Cambridge, UK) used were as follows: rabbit monoclonal anti-OPA1 antibody, rabbit monoclonal anti-Drp1 antibody, rabbit monoclonal anti-Mitofusin 2 antibody, mouse monoclonal anti-AMPK antibody, rabbit polyclonal anti-p-AMPK antibody, and rabbit polyclonal anti-beta actin antibody. After washing, the membranes were incubated for 1 h with the respective horseradish peroxidase-conjugated secondary antibodies at room temperature. Finally, the membranes were developed by enhanced chemiluminescence using a Bio-Rad imaging system (Bio-Rad, Hercules, CA, USA) [21].

### 2.17. Statistical Analysis

Results are expressed as the mean ± standard error of mean (SEM) of three independent experiments. Comparisons between two groups were performed by Student's *t*-test, while one-way ANOVA with Tukey's post hoc test was used for multigroup comparison. Statistical significance was set at *P* < 0.05.

## 3. Results

### 3.1. CE Attenuates I/R Injury-Induced Cardiac Dysfunction in Rats

**Figure 1:**
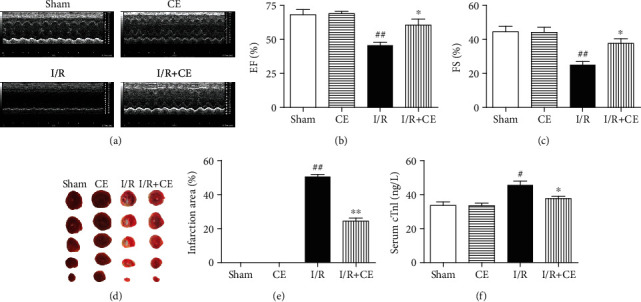
Effect of CE on I/R-induced cardiac dysfunction. (a) Representative trace of M-mode echocardiography performed 24 h after MI/R injury in a rat study. (b) Quantitative analysis of left ventricular ejection fraction (EF) and (c) fractional shortening (FS) using echocardiography. LVEF: left ventricular ejection fraction; FS: fractional shortening. (d) Representative images of TTC staining. Red-stained areas represent normal tissue, while unstained pale areas depict infarcted tissue. (e) Quantitative analysis of infarct size. (f) Measurement of serum cTnI levels. Data are presented as the mean ± SEM (*n* = 6). ^#^*P* < 0.05 vs. sham; ^##^*P* < 0.01 vs. sham; ^∗^*P* < 0.05 vs. I/R; ^∗∗^*P* < 0.01 vs. I/R.

To determine the protective effects of CE on cardiac function, M-mode echocardiography was used to measure cardiac parameters (Figure 1(a)). As shown in Figures 1(b) and 1(c), I/R severely impaired myocardial function, as demonstrated by significant decreases in EF and FS. However, CE treatment significantly improved I/R-induced cardiac dysfunction. An increase in myocardial infarct size was observed in MI/R rats compared with the sham group. However, CE treatment significantly decreased infarct size in comparison with the MI/R group (Figures 1(d) and 1(e)). In addition, the serum cTnI level, an indicator of myocardial injury, rapidly increased in MI/R hearts. CE treatment reduced the level of cTnI after MI/R (Figure 1(f)).

### 3.2. CE Inhibits I/R Injury-Induced Myocardial Mitochondrial Damage

**Figure 2:**
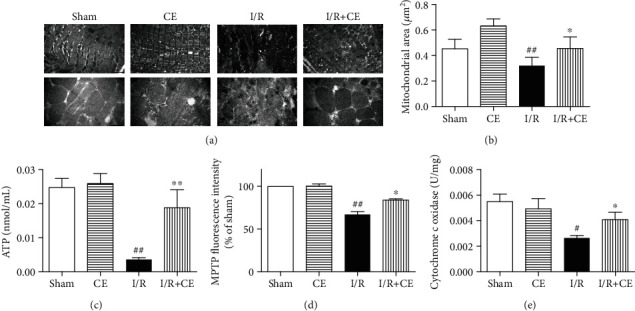
CE suppresses I/R-induced mitochondrial morphological damage and dysfunction. (a) Representative transmission electron microscopy (TEM) images of cardiac mitochondria. Scale bar (upper) = 2 *μ*m. Scale bar (lower) = 0.5 *μ*m. (b) Mean size of the mitochondria. (c) Myocardial ATP content. (d) Changes of mitochondrial permeability transition pore opening. (e) Cytochrome c oxidase activity. Data are presented as the mean ± SEM (*n* = 6). ^#^*P* < 0.05 vs. sham; ^##^*P* < 0.01 vs. sham; ^∗^*P* < 0.05 vs. I/R; ^∗∗^*P* < 0.01 vs. I/R.

The ultrastructure of cardiac mitochondria was evaluated by TEM. In the I/R-induced cardiomyocyte, the mitochondrial arrangement was irregular, with clusters of mitochondrial fragments and high diversity in shape and size. CE treatment significantly reduced these morphological changes (Figures 2(a) and 2(b)). Total ATP levels were significantly decreased in the I/R group, while CE significantly increased ATP levels (Figure 2(c)). Moreover, the mitochondrial permeability transition pore (MPTP) opening and cytochrome c oxidase activity explained the effects of CE on mitochondrial function. I/R significantly increased the MPTP opening and decreased the cytochrome c oxidase activity. However, CE effectively improved I/R-induced mitochondrial dysfunction (Figures 2(d) and 2(e)).

### 3.3. CE Decreases I/R-Induced Myocardial Apoptosis in Rats

**Figure 3:**
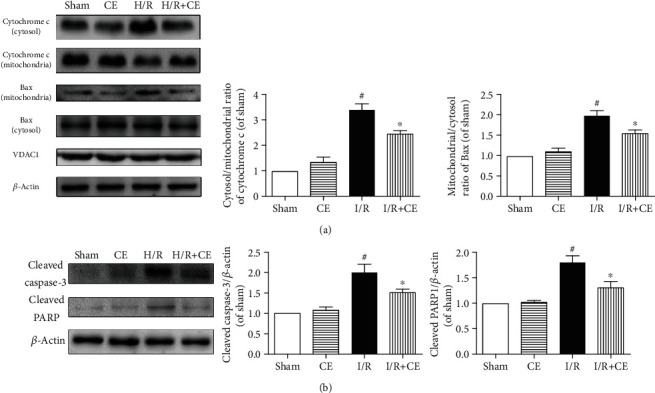
Effects of CE on apoptosis in I/R-injured hearts. (a) Changes in the expression of cytochrome c and Bax in the mitochondrial and cytosolic fractions and (b) changes in the expression of cleaved caspase-3 and cleaved PARP in isolated rat hearts. *β*-Actin expression was examined as a protein loading control. Data are presented as the mean ± SEM from three independent experiments. ^#^*P* < 0.05 vs. sham; ^∗^*P* < 0.05 vs. I/R.

Mitochondrial dysfunction is one of the main pathways of apoptosis and thus contributes to I/R injury [22]. CE decreased the expression of Bax in the mitochondrial fraction and inhibited mitochondrial cytochrome c release into the cytosol, which was induced by I/R (Figure 3(a)). In line with this, CE also significantly reduced the expression of cleaved caspase-3 and its substrate cleaved PARP compared with the I/R group (Figure 3(b)).

### 3.4. CE Attenuated H/R-Induced Injury and Apoptosis in Cardiomyocytes

**Figure 4:**
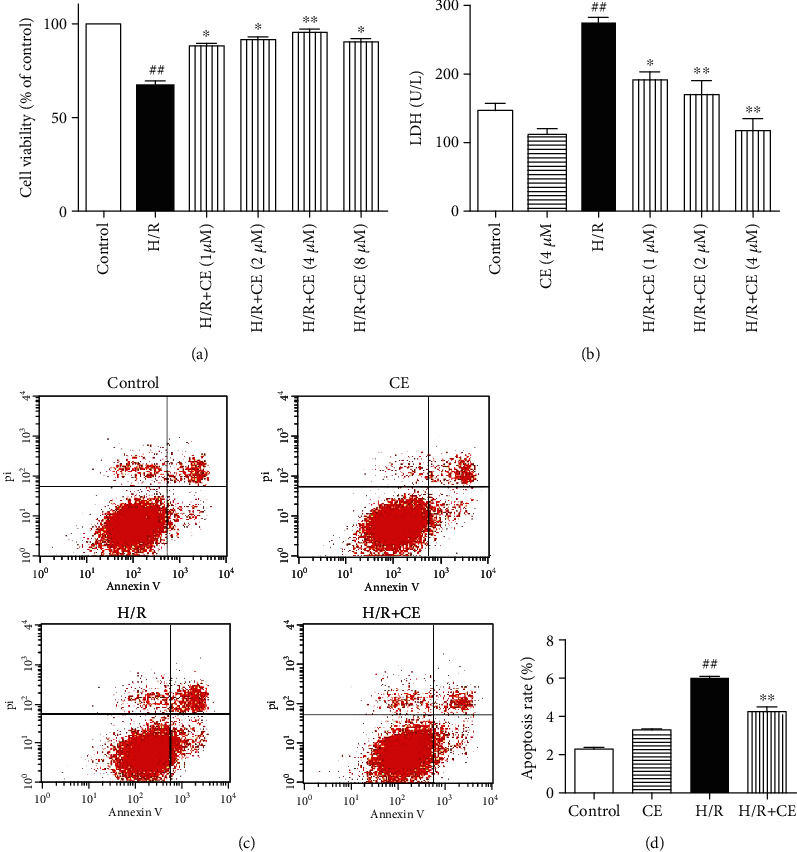
CE protects H9c2 cardiomyocytes against H/R-induced cell injury. (a) Cell viability was detected by the MTT assay. (b) Effects of CE on H/R-induced LDH leakage. (c) Apoptosis in H9c2 cardiomyocytes was analyzed by flow cytometry. (d) Quantitative analysis of the percentages of early apoptotic cells. The values are expressed as the mean ± SEM from three independent experiments. ^##^*P* < 0.01 vs. control; ^∗^*P* < 0.05 vs. H/R; ^∗∗^*P* < 0.01 vs. H/R.

The protective effects of CE against H/R-induced cell injury were detected by the MTT and LDH assays. As shown in Figure 4(a), CE dose-dependently attenuated H/R-induced reduction in cell viability. H/R-induced LDH release was also significantly decreased when the cells were pretreated with AsC in a dose-dependent manner (Figure 4(b)). CE at 4 *μ*M, which exhibited the most significant protective effect, was selected for further experiments.

The Annexin V-FITC/PI staining assay demonstrated that the number of early apoptotic cells significantly increased in H/R-treated H9c2 cardiomyocytes compared with the control group, while incubation with CE effectively alleviated H/R-induced early apoptosis (Figures 4(c) and 4(d)).

### 3.5. CE Regulates the Balance of Proteins Related to Mitochondrial Fission and Fusion in I/R Rats

**Figure 5:**
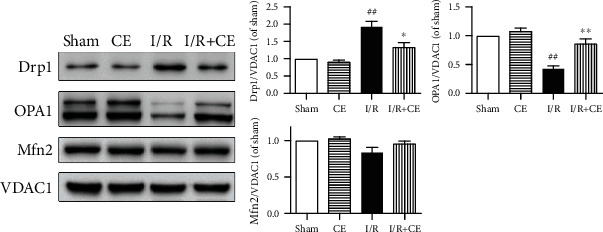
CE partially restored the expression of I/R-induced cardiac mitochondrial dynamic proteins. Representative immunoblots and western blot quantitative analysis of the mitochondrial dynamic protein expression of Drp1, OPA1, and Mfn2. VDAC1 expression was examined as a protein loading control. Data are presented as the mean ± SEM from three independent experiments. ^##^*P* < 0.01 vs. sham; ^∗^*P* < 0.05 vs. I/R; ^∗∗^*P* < 0.01 vs. I/R.

To determine the possible mechanisms through which CE protects the mitochondria against I/R injury, the expression levels of Drp1 (a protein that regulates fission events), OPA1 (which regulates mitochondrial inner membrane fusion), and Mfn1/2 (which controls outer membrane fusion) were monitored. As shown in Figure 5, in the I/R group, the level of Drp1 protein was significantly elevated, whereas Mfn1/2 and OPA1 were decreased compared with the sham group. CE treatment not only inhibited Drp1 expression but also recovered Mfn1/2 and OPA1 levels in comparison with the I/R group, indicating that CE improves the balance between fission and fusion following I/R injury.

### 3.6. Silencing of OPA1 Abolishes the Protective Effects of CE on Mitochondrial Homeostasis and Apoptosis in H/R-Treated Cardiomyocytes

**Figure 6:**
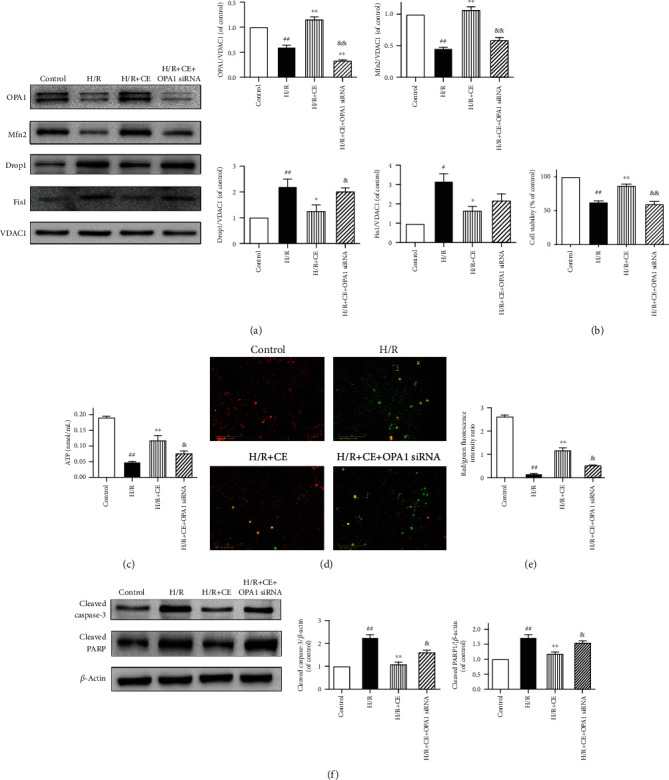
Effects of OPA1 on the protection of CE against H/R-stimulated mitochondrial injury and apoptosis. The siRNA against OPA1 was used to knock down the expression of OPA1. (a) Western blot assay and quantitative analyses of mitochondrial fission-related (Drp1 and Fis1) and fusion-related (OPA1 and Mfn2) proteins in H/R cardiomyocytes with CE and OPA1 siRNA pretreatment. (b) Effects of CE and OPA1 deficiency on cell viability in H/R-treated cardiomyocytes. (c) Effects of CE and OPA1 deficiency on ATP levels. (d) Effects of CE and OPA1 deficiency on mitochondrial transmembrane potential based on JC-1 staining. (e) Quantitative analysis of mitochondrial membrane potential. (f) Effects of CE and OPA1 deficiency on apoptotic proteins cleaved caspase-3 and cleaved PARP. *β*-Actin expression was examined as a protein loading control. Data are presented as the mean ± SEM from three independent experiments. ^##^*P* < 0.01 vs. control; ^∗^*P* < 0.05 vs. H/R; ^∗∗^*P* < 0.01 vs. H/R; ^&^*P* < 0.05 vs. H/R+CE; ^&&^*P* < 0.01 vs. H/R+CE.

To determine the role of OPA1 in the beneficial effects of CE in cardiomyocytes, we used small interfering RNA (siRNA) to silence OPA1 in cardiomyocytes. We found that CE treatment enhanced the profusion proteins (OPA1 and Mfn2) and reduced the levels of profission proteins (Drp1 and Fis1). However, the loss of OPA1 abolished the regulatory effects of CE on mitochondrial fusion and fission (Figure 6(a)). As shown in Figures 6(b) and 6(c), OPA1 ablation reversed the protection against H/R injury by decreasing cell viability and ATP production. We then measured mitochondrial membrane potential (*ΔΨ*m) in cardiomyocytes. As shown in Figures 6(d) and 6(e), H/R injury impaired *ΔΨ*m, while CE treatment improved the stability of *ΔΨ*m. Notably, the effects of CE were negated when OPA1 was knocked down. Additionally, cardiomyocyte apoptosis was detected by analyzing the expression of cleaved caspase-3 and PARP (Figure 6(f)). Compared to the control group, the presence of H/R-activated caspase-3 and PARP could be inhibited by CE. However, OPA1 silencing abrogated the antiapoptotic effects of CE on cardiomyocytes.

### 3.7. CE Activates AMPK Following Myocardial I/R Injury

**Figure 7:**
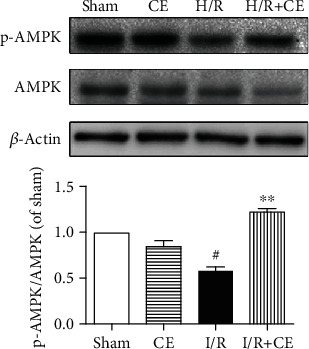
Effects of CE on AMPK activation. Representative western blot and quantitative analyses of protein expression of p-AMPK, AMPK, and *β*-actin. The values are expressed as the mean ± SEM from three independent experiments. ^#^*P* < 0.05 vs. sham; ^∗∗^*P* < 0.01 vs. I/R.

To determine whether CE protects the heart against myocardial I/R injury through the AMPK signaling pathway, we examined the effect of CE on the cardiac AMPK signaling pathway.  Figure 7 shows that CE significantly increased the phosphorylation of AMPK compared with the I/R group, whereas no change was detected in the levels of total AMPK.

### 3.8. CE Regulates OPA1 via the AMPK Signaling Pathway

**Figure 8:**
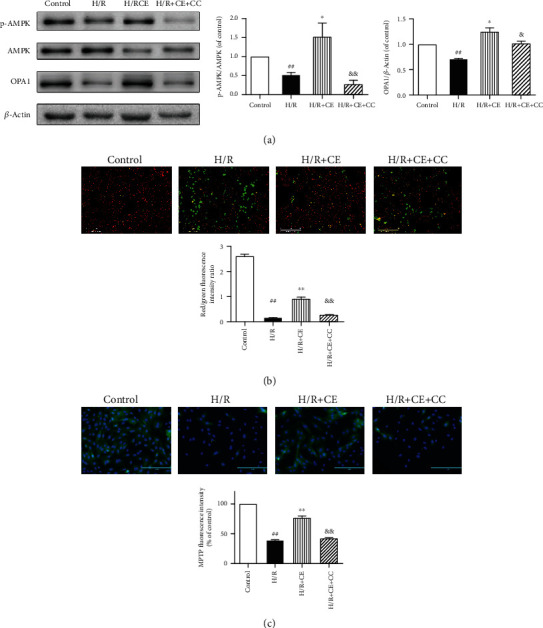
CE improves OPA1 expression by activating the AMPK pathway. (a) Western blotting was used to analyze the changes in OPA1 and AMPK in response to CE and/or CC treatment. Compound C (CC), an AMPK pathway inhibitor, was added to the cardiomyocytes. *β*-Actin expression was examined as a protein loading control. (b) Mitochondrial membrane potential. (c) Changes of mitochondrial permeability transition pore opening. Data are presented as the mean ± SEM from three independent experiments. ^##^*P* < 0.01 vs. control; ^∗^*P* < 0.05 vs. H/R; ^∗∗^*P* < 0.01 vs. H/R; ^&^*P* < 0.05 vs. H/R+CE; ^&&^*P* < 0.01 vs. H/R+CE.

Western blotting analysis demonstrated that CE treatment upregulated the levels of p-AMPK and OPA1 in H/R-treated cardiomyocytes. However, the inhibition of AMPK by compound C (CC) caused a decline in OPA1 expression (Figure 8(a)), which suggests that OPA1 expression was modulated by the AMPK signaling pathway.

To confirm whether AMPK is also involved in cardiomyocyte survival and mitochondrial homeostasis, we analyzed cell viability, mitochondrial membrane potential, and MPTP opening. As shown in Figure 8(b), the H/R-induced loss of *ΔΨ*m was also suppressed by CE, and this beneficial effect was inhibited after blockade of the AMPK pathway. Moreover, the H/R group displayed calcein fluorescence loss, which indicated that the MPTP was opening. CE treatment reduced the MPTP opening induced by H/R, but this effect was not observed following treatment with CC (Figure 8(c)).

## 4. Discussion

In the present study, several important observations were made. First, we further confirmed that CE significantly preserved cardiac function in rats subjected to MI/R injury as well as increased the viability of cardiomyocytes under H/R. Second, CE improved mitochondrial function and inhibited apoptosis by enhancing OPA1-related mitochondrial fusion during I/R injury. Third, we found that CE increased AMPK phosphorylation, while AMPK inhibitor compound C mitigated the positive effects of CE on mitochondrial dynamics and apoptosis. Taken together, our study provides direct evidence that CE enhances mitochondrial function and exerts powerful protection against myocardial I/R damage via the AMPK-OPA1 pathway.

Mitochondrial dysfunction plays a crucial role in MI/R injury; this includes decreasing mitochondrial metabolic enzymes and ATP content and opening the mitochondrial permeability transition pore (MPTP) which contributes to mitochondrial Ca^+^ overload and oxidative bursts, leading to apoptosis or necrosis [22]. Given the pivotal role of mitochondria in the heart, we determined mitochondrial function during MIRI. Consistent with the literature [23], our study showed that I/R destroyed mitochondrial morphology, resulting in mitochondrial swelling and crista morphological disorder. Meanwhile, ATP production declined, MMP reduced, and apoptotic expression increased during MIRI. However, CE attenuated I/R-induced mitochondrial ultrastructural damage and maintained mitochondrial function.

Mitochondria are dynamic organelles continually undergoing fission and fusion, a process referred to as mitochondrial dynamics [8]. Mitochondrial function depends heavily on the alteration of mitochondrial ultrastructure and morphology, which are tightly linked to mitochondrial fission and fusion. Mitochondrial dynamics are regulated by specific fusion and fission proteins [24]. The proteins that play roles in helping fusion of the outer and inner membranes are Mitofusin 2 (Mfn2) and optic atrophy 1 (OPA1), respectively, whereas the protein that plays a role in membrane constriction during fission is dynamin-related protein 1 (Drp1). There is abundant evidence that the imbalance of mitochondrial fusion and fission results in abnormal mitochondrial structure and disrupted mitochondrial function during MI/R injury [24]. Inhibition of the expression of mitochondrial fission-associated proteins, such as dynamin-related protein 1 (Drp1) and fission 1 (Fis1), has been shown to reduce infarct size and improve left ventricular dysfunction during MIRI [25, 26]. Concomitantly, promoting mitochondrial fusion can ameliorate abnormal mitochondrial morphology, increase ATP production, and reduce mitochondrial-derived apoptosis in MIRI [10]. In recent years, increasing evidence has shown that OPA1, which plays a crucial role in regulating mitochondrial fusion, has protective effects in various cardiac diseases, especially in I/R [24, 27]. Elevating the expression of OPA1 by drug preconditioning or overexpression of OPA1 could improve the morphology and function of mitochondria and reduce myocardial injury [12, 13, 28]. However, conditioned OPA1 knockdown aggravates mitochondrial dysfunction and cell damage [13, 29]. In the present study, we found that the expression of mitochondrial fission proteins Drp1 and Fis1 was remarkably increased, whereas that of fusion proteins OPA1 and Mfn1/2 was decreased in the I/R group when compared to the sham group. In contrast, CE rectified I/R-induced turbulence in mitochondrial dynamics, subsequently improving mitochondrial dysfunction and decreasing cardiomyocyte apoptosis. However, these effects of CE were abolished by OPA1 deletion, suggesting that OPA1-related mitochondrial fusion is related to the cardioprotection of CE against MIRI.

Moreover, we found that OPA1 expression increased by CE was associated with AMPK phosphorylation. AMPK, a sensor of cellular energy shortage, plays a key role in regulating cell survival and death in response to pathological stress [30]. Recent studies have shown that OPA1-related mitochondrial activity and dynamics are regulated by AMPK [31]. Pharmacological activation of AMPK prevents mitochondrial dysfunction and cardiomyocyte death by promoting OPA1-related mitochondrial fusion during cardiac I/R injury [13]. As expected, our findings illustrated that CE activated AMPK in I/R-induced myocardial damage. Meanwhile, the effects of CE on promoting OPA1 expression and mitochondrial function were repressed after the inhibition of AMPK by CC, which suggests that AMPK is necessary for CE-mediated protection of mitochondrial dynamics and function during MI/R injury.

## 5. Conclusions

Our findings demonstrated that CE alleviated MI/R-induced mitochondrial dysfunction and mitochondrial dynamic imbalance by enhancing OPA1-related mitochondrial fusion via the activation of AMPK signaling pathways. This study unveiled the molecular mechanisms responsible for the beneficial effects of CE and provided a promising therapeutic strategy for the treatment of myocardial I/R injury.

## Data Availability

The data used to support the findings of this study are available from the corresponding authors upon request.

## Abbreviations

AMPK: AMP-activated protein kinase
AS: *Aralia elata* (Miq.) Seem.
CE: Calenduloside E
H/R: Hypoxia/reoxygenation
I/R: Ischemia/reperfusion
LDH: Lactate dehydrogenase
MI/R: Myocardial ischemia/reperfusion
MIRI: Myocardial ischemia/reperfusion injury
MPTP: Mitochondrial permeability transition pore
CC: Compound C.

## References

[1] Bromage D. I., Pickard J. M., Rossello X. (2017). Remote ischaemic conditioning reduces infarct size in animal *in vivo* models of ischaemia-reperfusion injury: a systematic review and meta-analysis. *Cardiovascular Research*.

[2] Wang M., Xu X., Xu H. (2014). Effect of the total saponins of *Aralia elata* (Miq) Seem on cardiac contractile function and intracellular calcium cycling regulation. *Journal of Ethnopharmacology*.

[3] Shi M., Yang Y., Sun Y. (2014). Pharmacokinetic study of calenduloside E and its active metabolite oleanolic acid in beagle dog using liquid chromatography–tandem mass spectrometry. *Journal of Chromatography B*.

[4] Gui-Bo S., Hui-Bo X., Fu-Chun W., Tao D., Xiao-Bo S. (2006). Anti-arrhythmic effect of deglucose-chikusetsu-saponin IVa. *Chinese Journal of Pharmacology and Toxicology*.

[5] Tian Y., Du Y. Y., Shang H. (2017). Calenduloside E analogues protecting H9c2 cardiomyocytes against H_2_O_2_-induced apoptosis: design, synthesis and biological evaluation. *Frontiers in Pharmacology*.

[6] Wang S., Tian Y., Zhang J. Y. (2018). Targets fishing and identification of calenduloside E as Hsp90AB1: design, synthesis, and evaluation of clickable activity-based probe. *Frontiers in Pharmacology*.

[7] Tian Y., Wang S., Shang H. (2017). The proteomic profiling of calenduloside E targets in HUVEC: design, synthesis and application of biotinylated probe BCEA. *RSC Advances*.

[8] Hernandez-Resendiz S., Prunier F., Girao H., Dorn G., Hausenloy D. J., EU‐CARDIOPROTECTION COST Action (CA16225) (2020). Targeting mitochondrial fusion and fission proteins for cardioprotection. *Journal of Cellular and Molecular Medicine*.

[9] Cooper H. A., Eguchi S. (2018). Inhibition of mitochondrial fission as a novel therapeutic strategy to reduce mortality upon myocardial infarction. *Clinical Science*.

[10] Maneechote C., Palee S., Kerdphoo S., Jaiwongkam T., Chattipakorn S. C., Chattipakorn N. (2019). Balancing mitochondrial dynamics via increasing mitochondrial fusion attenuates infarct size and left ventricular dysfunction in rats with cardiac ischemia/reperfusion injury. *Clinical Science*.

[11] Lee H., Yoon Y. (2018). Mitochondrial membrane dynamics—functional positioning of OPA1. *Antioxidants*.

[12] Wang K., Liu Z., Zhao M. (2020). *κ*-Opioid receptor activation promotes mitochondrial fusion and enhances myocardial resistance to ischemia and reperfusion injury via STAT3-OPA1 pathway. *European Journal of Pharmacology*.

[13] Zhang Y., Wang Y., Xu J. (2019). Melatonin attenuates myocardial ischemia‐reperfusion injury via improving mitochondrial fusion/mitophagy and activating the AMPK‐OPA1 signaling pathways. *Journal of Pineal Research*.

[14] Chen W. R., Zhou Y. J., Yang J. Q., Liu F., Wu X. P., Sha Y. (2020). Melatonin attenuates calcium deposition from vascular smooth muscle cells by activating mitochondrial fusion and mitophagy via an AMPK/OPA1 signaling pathway. *Oxidative Medicine and Cellular Longevity*.

[15] He S., Zhang C., Zhou P. (2019). Herb-induced liver injury: phylogenetic relationship, structure-toxicity relationship, and herb-ingredient network analysis. *International Journal of Molecular Sciences*.

[16] Xu L. J., Chen R. C., Ma X. Y., Zhu Y., Sun G. B., Sun X. B. (2020). Scutellarin protects against myocardial ischemia-reperfusion injury by suppressing NLRP3 inflammasome activation. *Phytomedicine*.

[17] Zhang J. Y., Wang M., Wang R. Y. (2018). Salvianolic acid A ameliorates arsenic trioxide-induced cardiotoxicity through decreasing cardiac mitochondrial injury and promotes its anticancer activity. *Frontiers in Pharmacology*.

[18] Schneider C. A., Rasband W. S., Eliceiri K. W. (2012). NIH image to ImageJ: 25 years of image analysis. *Nature Methods*.

[19] Wang M., Sun G. B., Du Y. Y. (2017). Myricitrin protects cardiomyocytes from hypoxia/reoxygenation injury: involvement of heat shock protein 90. *Frontiers in Pharmacology*.

[20] Ye J. X., Wang M., Wang R. Y. (2020). Hydroxysafflor yellow A inhibits hypoxia/reoxygenation-induced cardiomyocyte injury via regulating the AMPK/NLRP3 inflammasome pathway. *International Immunopharmacology*.

[21] Wang M., Tian Y., Du Y. Y. (2017). Protective effects of Araloside C against myocardial ischaemia/reperfusion injury: potential involvement of heat shock protein 90. *Journal of Cellular and Molecular Medicine*.

[22] Li Y., Chen B., Yang X. (2019). S100a8/a9 signaling causes mitochondrial dysfunction and cardiomyocyte death in response to ischemic/reperfusion injury. *Circulation*.

[23] Xue R. Q., Sun L., Yu X. J., Li D. L., Zang W. J. (2017). Vagal nerve stimulation improves mitochondrial dynamicsviaan M3receptor/CaMKK*β*/AMPK pathway in isoproterenol-induced myocardial ischaemia. *Journal of Cellular and Molecular Medicine*.

[24] Maneechote C., Palee S., Chattipakorn S. C., Chattipakorn N. (2017). Roles of mitochondrial dynamics modulators in cardiac ischaemia/reperfusion injury. *Journal of Cellular and Molecular Medicine*.

[25] Ding M., Dong Q., Liu Z. (2017). Inhibition of dynamin-related protein 1 protects against myocardial ischemia-reperfusion injury in diabetic mice. *Cardiovascular Diabetology*.

[26] Jin Q., Li R., Hu N. (2018). DUSP1 alleviates cardiac ischemia/reperfusion injury by suppressing the Mff-required mitochondrial fission and Bnip3-related mitophagy via the JNK pathways. *Redox Biology*.

[27] Burke N., Hall A. R., Hausenloy D. J. (2015). OPA1 in cardiovascular health and disease. *Current Drug Targets*.

[28] Guan L., Che Z., Meng X. (2019). MCU up-regulation contributes to myocardial ischemia-reperfusion injury through calpain/OPA-1-mediated mitochondrial fusion/mitophagy inhibition. *Journal of Cellular and Molecular Medicine*.

[29] Le Page S., Niro M., Fauconnier J. (2016). Increase in cardiac ischemia-reperfusion injuries in Opa1+/- mouse model. *PLoS One*.

[30] Steinberg G. R., Carling D. (2019). AMP-activated protein kinase: the current landscape for drug development. *Nature Reviews Drug Discovery*.

[31] Herzig S., Shaw R. J. (2018). AMPK: guardian of metabolism and mitochondrial homeostasis. *Nature Reviews Molecular Cell Biology*.

